# *Perlesta ephelida*, a new Nearctic stonefly species (Plecoptera, Perlidae)

**DOI:** 10.3897/zookeys.194.2972

**Published:** 2012-05-17

**Authors:** Scott A. Grubbs, R. Edward DeWalt

**Affiliations:** 1 Department of Biology and Center for Biodiversity Studies, Western Kentucky University, Bowling Green, KY 42101 USA; 2 University of Illinois, Prairie Research Institute, Illinois Natural History Survey, 1816 S Oak St., Champaign, IL 61820 USA

**Keywords:** *Perlesta*, Perlidae, Plecoptera, stonefly, new species

## Abstract

A new Nearctic species of Perlidae (Insecta, Plecoptera), *Perlesta ephelida* **sp. n.**, is described from the male, female, and egg stages. This species has been previously reported as, or confused with, *Perlesta shubuta* Stark from several central and eastern U.S. states. *Perlesta ephelida* is distinctive from *Perlesta shubuta* and other regional Nearctic congeners mainly according to male genitalic and egg characteristics. *Perlesta ephelida* is a widely-distributed eastern Nearctic species, whereas *Perlesta shubuta* appears to be restricted to a narrow latitudinal belt in the Gulf Coast region from Louisiana east conservatively to the Florida panhandle. The egg of *Perlesta shubuta* is depicted with scanning electron microscopy for the first time.

## Introduction

*Perlesta shubuta* Stark (Plecoptera: Perlidae) is a small-bodied, darkly-pigmented stonefly described from southern Mississippi (Stark 1989). This species has since been reported from Alabama (Graves and Ward 2011), Arkansas, Missouri and Oklahoma (Poulton and Stewart 1991), Florida (Ray et al. 2012), Illinois (DeWalt et al. 2001), Indiana (DeWalt and Grubbs 2011), Iowa (Heimdal et al. 2004), Kentucky (Tarter et al. 2006), Michigan (Grubbs and Bright 2001), North Carolina (Kondratieff et al. 2011), and West Virginia (Tarter and Nelson 2010). In addition, there are unpublished records for Florida (Pescador et al. 2000) and South Carolina (Pescador et al. 2004) as well as material that resemble *Perlesta shubuta* from several additional central and eastern U.S. states obtained via loans or collected by both authors since the mid-1990s.

The male aedeagus of *Perlesta shubuta* bears a thumb-shaped caecum, a common feature for the genus (Stark 2004) and part of the reason why this species is somewhat difficult to identify with complete certainty. The female subgenital plate is likewise similar to other species. Although the morphology of the egg appears to be distinctive (Stark 1989) it has yet to be studied with scanning electron microscopy to reveal fine structural details of the chorion.

The type locality for *Perlesta shubuta* is a sandy-bottom headwater stream. Poulton and Stewart (1991), DeWalt et al. (2001), and Heimdal et al. (2004), however, reported *Perlesta shubuta* from large stream systems. DeWalt and Grubbs (2011) suggested that the Illinois and Indiana specimens they tentatively determined as *Perlesta shubuta* may represent a new species. Stark (1989) and Poulton and Stewart (1991) both commented on “extremely freckled” and “speckled” nymphs, respectively, from the Ozark and Ouachita Mountain region, and the latter authors stated that “…our specimens are tentatively placed under *Perlesta shubuta* and may represent an undescribed species”.

Our material that we only tentatively identified as *Perlesta shubuta* from throughout the central and eastern U.S. has been obtained mainly from larger systems, including low elevation Appalachian Mountain rivers in Alabama, Maryland and West Virginia, and sandy-bottom lowland rivers in Illinois, Indiana and Michigan. Associated nymphs and exuviae obtained through rearings bear a head mask that is freckled to a much greater degree compared to regional congeners. Because of the uncertainty regarding the identity of *Perlesta shubuta* from localities other than at/near the type locality in southern Mississippi, a comparative study was undertaken to examine available material from across the central and eastern U.S.

## Materials and methods

Fresh specimens of *Perlesta shubuta* were obtained from eastern Mississippi and western Alabama in May 2011. A visit to the type locality was unsuccessful. Recently collected material of *Perlesta shubuta*, in addition to all specimens determined tentatively as *Perlesta shubuta*, was reexamined with light microscopy to study fine details of the paraprocts, dorsal and lateral aedeagal spinule patterns, and the subgenital plate. Material were obtained from collections from the Bill P. Stark Collection, Mississippi College, Mississippi (BPS), Brigham Young University, Monte L. Bean Museum, Provo, Utah (BYU), Canadian National Collection, Ottawa (CNC), Illinois Natural History Survey, insect collection series, Champaign, Illinois (INHS), Illinois Natural History Survey, Plecoptera catalog series, Champaign, Illinois (INHSPle), A.J. Cook Arthropod Research Collection, Michigan State University, East Lansing, Michigan (MSUC), Ohio Biological Survey, Columbus (OBS), Purdue University Research Collections, West Lafayette, Indiana (PURC), the Royal Ontario Museum, Toronto (ROME), University of Michigan Zoology Collection, Ann Arbor, Michigan (UMMZ), Scott A. Grubbs Collection, Western Kentucky University, Bowling Green, Kentucky (WKU), and University of Wisconsin Entomological Research Center, Madison, Wisconsin (WIRC). All material examined is stored in ethyl alcohol.

Scanning electron micrographs (SEM) of eggs and male dorsal terminalia were prepared from the Alabama and Mississippi samples of *Perlesta shubuta*. Egg and male SEMs were also prepared for several, tentatively-identified *Perlesta shubuta* specimens from a broad geographic coverage of the central and eastern U.S.

## Taxonomy

### Perlesta shubuta Stark

http://species-id.net/wiki/Perlesta_shubuta

Figs 8 22–24

Perlesta shubuta Stark 1989: 282 [Type locality: USA, Mississippi, Simpson Co., Mill Creek; National Museum of Natural History; male]. DeWalt et al. 2011 [distribution].

#### Material examined.

**USA: Alabama:** Choctaw Co., Bogue Chitto Creek, 20 km NW Butler, 32.1872, -88.3969, 17.v.2011, S.A. Grubbs, ♂, 2♀ (WKU); tributary to Yantley Creek, 13 km NW Cromwell, 32.3004, -88.3837, 17.v.2011, S.A. Grubbs, ♂ (WKU); Sumter Co., Brockway Creek, 8 km SE Ward, 32.3369, -88.1977, 17.v.2011, S.A. Grubbs, ♂, ♀ (WKU). **Louisiana:** Natchitoches Parish, Kisatchie Bayou, Kisatchie Bayou Campground, Forest Rd. 366, 31.4416, -93.0893, 18.v.1992, at light, R.E. DeWalt, 2♂, ♀ (INHS), same but 9.vi.1992 (reared), R.E. DeWalt, 3♂, ♀ (INHS). **Mississippi:** Clarke Co., tributary to Long Creek, 20 km E Enterprise, 32.1673, -88.6260, 16.v.2011, S.A. Grubbs, ♂, ♀ (WKU); Rolling Creek, 8 km E Stonewell, 32.1470, -88.7076, 16.v.2011, S.A. Grubbs, 2♂, 2♀ (WKU); Simpson Co., Mill Creek, 14 May 1981, B.P. Stark, ♂ (Paratypes – BPS).

#### Egg.

Oblong (Fig. 22). Collar wide but short, highly-infolded, and flanged distally (Figs 23–24). Chorion covered completely by shallow depressions that are visible at higher magnification (1000×; Fig. 23–24).

#### Comments.

If the aedeagus is extruded fully, the combination of the updated taxonomic key provided in Stark (2004) and descriptions of *Perlesta shubuta* by Stark (1989, 2004) are sufficient to identify males of this species. In absence of eggs, however, females cannot be reliably identified to species. The distally-flanged, short egg collar is similar only to *Perlesta nelsoni* Stark, 1989 (see Grubbs 2005, Fig. 7), a species known from southern and central Appalachian drainages from Tennessee north to Pennsylvania and northward into New York (Stark 2004, Kondratieff and Myers 2011). The line drawing of *Perlesta shubuta* provided by Stark (see Stark 1989, Fig. 93) does not show the flanged egg collar.

### Perlesta ephelida

Grubbs & DeWalt sp. n.

urn:lsid:zoobank.org:act:C5983849-EE20-4A42-BEB6-B5588AA1500A

http://species-id.net/wiki/Perlesta_ephelida

Figs 1 –7 9 –21

#### Material examined.

**Holotype:** male, in alcohol, **USA: Kentucky:** Warren Co., Trammel Fork, Drakes Creek, Alvaton, 36.8704, -86.3683, 2.vi.1999, S.A. Grubbs (INHS).

#### Paratypes. 

**USA: Alabama:** Clay Co., Enitachopco Creek, CR 35, 9 km ESE Millersville, 33.1602, -85.8352, 18.v.2008, S.A. Grubbs , 3♂, 2♀ (WKU). **Illinois:** Winnebago Co., Sugar River, Sugar River Forest Preserve, 42.4604, -89.2399, 22.vi.2010, R.E. DeWalt, E.W. Hernandez and M.M. Brown, 15♂ (INHS-516704). **Indiana:** Bartholomew Co., East Fork White River, Azalia Bridge, 1.5 km SW Azalia, 39.0849, -85.8598, 11.vi.2000, S.A. Grubbs, 4♂, 3♀ (WKU), same but 9.vii.2008, S.A. Grubbs,♀ (WKU); Pulaski Co., Tippecanoe River, 8 km NNE Winamac, 41.1316, -86.5880, 28.vi.2006, R.E. DeWalt, 4♂, 3♀ (INHS-164863); Steuben Co., Pigeon River, Rte. 327, 14 km N Helmer, 41.6513, -85.1744, 23.vi.2006, S.A. Grubbs, 3♂, 4♀, 2 nymphs (WKU). **Kentucky:** Warren Co., same data as Holotype but 24.v.1999, S.A. Grubbs, ♂, 3♀ (WKU), same but 30.v.1999, S.A. Grubbs, ♂ (WKU), same but 19.vi.1999, S.A. Grubbs, ♀ (WKU), same but 20.vi.1999 (reared), S.A. Grubbs, ♀, nymph, exuvia (WKU), same but 22.vi.1999 (reared), S.A. Grubbs, ♀, exuvia (WKU), same but 26.vi.1999, S.A. Grubbs, ♀ (WKU), same but 29.vi.1999, S.A. Grubbs, ♀ (WKU), same but 2.vii.1999, S.A. Grubbs, ♂, ♀ (WKU), same but 9.vii.1999, S.A. Grubbs, ♀ (WKU), same but 20.v.2001, S.A. Grubbs, ♀ (WKU), same but 30.v.2011, S.A. Grubbs, ♂, ♀ (WKU). **Maryland:** Washington Co., Licking Creek, 1 km N Pectonville, 39.6760, -78.0430, 3.viii.1996, S.A. Grubbs, 7♂ (WKU), same but 6.viii.1996, S.A. Grubbs, ♂ (WKU), same but 12.vii.1997, S.A. Grubbs, 25♂, 69♀ (WKU). **Michigan:** Calhoun Co., South Branch Kalamazoo River, M-60, Homer, 42.1469, -84.8023, 25.vii.2006, S.A. Grubbs, 6♂, 8♀ (WKU); Ionia Co., Flat River, 2 km N Belding, Flat River State Game Area, 43.1197, -85.2243, 21.vi.2006, S.A. Grubbs, 3♂, 2♀, 4 nymphs (WKU); St. Joseph Co., Rocky River, 1 km SW Moore Park at Floating Bridge Rd., 42.0056, -85.6461, 9.vi.2010, R.E. DeWalt, M. Pessino and E.W. Hernandez, 6♂, 3♀ (INHS-549882). **Missouri:** Oregon Co., Eleven Point River, Rte.160, Riverton Access, 36.6489, -91,2004, 27.vi.2011, S.A. Grubbs, 2♂, ♀ (WKU). **Ohio:** Portage Co., Cuyahoga River, 6 km E Streetsboro Coits Rd., 41.2493, -81.2694, 25.vi.2006, R.E. DeWalt, 11♂, 14♀ (INHS-163781). **Wisconsin:** Sawyer Co., Namekagon River, NE Hayward, public canoe put-in, 46.0720, -91.4170, 25.vi.2010, R.E. DeWalt, E. Hernandez and M.M. Brown, 5♂, 6♀ (INHS-516414).

#### Additional material examined.

**CANADA:** Flood River, [5 km S Atikokan], 48.7107, -91.5968, ROM Field Party, 3.vii.1984, 4 nymphs (ROM-9496); Ottawa River, Remic Rapids Champlain Bridge, 45.4078, -75.7544, 3.vii.1935, F. P. Ide, 2 nymphs (CNC-1282). **USA, Alabama:** Calhoun Co., Terrapin Creek, U.S. 278 at CR 33, 4 km NE Piedmont, 33.9550, -85.5712, 17.v.2008, S.A. Grubbs, ♂, ♀ (WKU); Cleburne Co., Tallapoosa River, CR 72, 21 km ENE Heflin, 33.7140, -85.3733, 8.vi.2007, S.A. Grubbs, ♀ (WKU); Talladega Co., Cheaha Creek, CR 005, 12 km NE Talladega, 33.5319, -86.0415, 18.v.2008, S.A. Grubbs, 5♀ (WKU); Talladega Creek, CR 303, 6 km S Talladega, 33.3826, -86.0790, 18.v.2008, S.A. Grubbs, 3♀ (WKU); Tallapoosa Co., Hillabee Creek, 15 km NE Alexander City, 33.0657, -85.8798, 18.v.2008, S.A. Grubbs, ♀ (WKU). **Arkansas:** Lawrence Co., Spring River, 1 km S Ravenden, 36.2246, -91.2505, 27.vi.2011, S.A. Grubbs, 2♀ (WKU); Sharp Co., Martins Creek, 5 km NE Wilford, 36.2722, -91.3326, 27.vi.2011, S.A. Grubbs, ♂ (WKU). **Illinois:** Clark Co., Big Creek, 6.5 km NNW Marshall, 39.4480, -87.7170, 21.vi.1993, D.W. Webb, 3♂, 2♀ (INHSPle-2978); Franklin Co., Big Muddy River, Zeigler, 37.8914, -89.0199, 6.viii.1976, S. Herbert, ♂ (INHS-487359). **Indiana:** Allen Co., Cedar Creek, 1 km W Cedar Canyons at Auburn Rd., 41.2351, -85.1027, 27.vi.2007, R.E. DeWalt, 14♂ (INHS-454376); Cass Co., Eel River, 1 km NE Adamsboro, along CR 600E, 40.7970, -86.2571, 24.v.2006, R.E. DeWalt, ♂, nymph (INHS-164562); Elkhart Co., Little Elkhart River, 5 km E Bristol, Bonnieville Mill County Park, 41.7192, -85.7630, 23.vi.2006, S.A. Grubbs, ♂, 3♀ (WKU); Franklin Co., West Fork Whitewater River, nr. U.S. 52, 1 km W Metamora, 39.4550, -85.1562, 6.vi.2009, S.A. Grubbs, ♀ (WKU); Johnson Co., Sugar Creek, 4.2 km NW Edinburgh, 39.3821, -85.9978, 21.vi.2006, R.E. DeWalt, ♂ (INHS-163722); Kosciusko Co., Cherry Creek, Winona Lake, 41.2270, -85.8220, 30.vi.1947, F, E. Mockford, ♂ (CNC-1288); Monroe Co., Bloomington, 39.1653, -86.5264, 9.vii.1938, Sherrill, ♀ (CNC-1305); Morgan Co., 39.6206, -86.2819, 28.vi.1933, Musgrave, ♀ (PURC-4261); Newton Co., Beaver Lake Ditch, 0.8 km NE Conrad, 41.1073, -87.4373, 8.vii.1998, E. H. Metzler, ♀ (OBS-2910); Starke Co., Kankakee River, 0.25 km N English Lake, 41.2720, -86.8246, 27.vi.2006, R.E. DeWalt, ♀ (INHS-164854); Tippecanoe Co., Wabash River, Lafayette, 40.4202, -86.8971, 16.vii.1923, G. M. Sterrett, ♂ (CNC-1834). **Iowa:** Buchanon Co., Lime Creek, 3.2 km NE Brandon, 42.3296, -91.9805, 8.vi.2000, D. Heimdal, ♂, ♀ (INHS-36090). **Kentucky:** Allen Co., Long Creek, Rte. 100, 10 km SE Scottsville, Barren River Lake WMA, 36.6965, -86.0469, 14.vi.2001, S.A. Grubbs and J.M. Taylor, ♂, 15♀ (WKU); Bell Co., Cumberland River, NE of Pineville along US-119, 36.7226, -83.6398, 7.vi.2007, R.E. DeWalt and L. Fennema, 5♂, ♀ ( INHS-454363); Green Co., Little Brush Creek, 5 km WNW Summersville, 37.3344, -85.5913, 3.vi.2006 (reared), S.A. Grubbs, ♂ (WKU); Brush Creek, Rte. 61, 10 km NW Summersville, 37.3909, -85.6105, 3.vi.2006 (reared), S.A. Grubbs, ♀ (WKU); Warren Co., Middle Fork Drakes Creek, 19 km NE Franklin, 36.8187, -86.3944, 10.vi.1999, S.A. Grubbs, ♂, ♀ (WKU); at confluence of Barren River and Green River, 15 km NW Richardsville, 37.1809, -86.6232, 4.vi.1999, S.A. Grubbs, ♂ (WKU); **Maryland:** Allegany Co., Flintstone Creek, Flintstone, 14.viii.1995, 39.7079, -78.5664, S. A. Grubbs, ♀ (WKU), same but 4.vii.1996, S.A. Grubbs, 9♂, 4♀ (WKU), same but 2.viii.1996, S. A. Grubbs, ♂, 3♀ (WKU); Sideling Hill Creek, Scenic U.S. 40, nr. Bellegrove, 39.7044, -78.3284, 3.viii.1996, S.A. Grubbs, 7♀ (WKU), same but 23.vi.1997, S.A. Grubbs, 3♀ (WKU), same but 14.vii.1998, S.A. Grubbs, 3♂ (WKU); Sideling Hill Creek, 1 km NE Bellegrove, 39.7084, -78.3313, 4.vii.1996, S.A. Grubbs, ♂, 6♀ (WKU); Town Creek, 5 km NE Oldtown, Green Ridge State Forest, 39.5744, -78.5468, 4.vii.1996, S.A. Grubbs, 2♂, 10♀ (WKU), same but 8.vi.1998, S.A. Grubbs, 3♂, 2♀ (WKU); Harford Co., Deer Creek, 7 mi NW Bel Air, Rocks State Park, 39.6314, -76.4052, 12.vii.1998, S.A. Grubbs, ♂, 2♀ (WKU); Washington Co., Conococheague Creek, 1 km E Fairview, 39.7163, -77.8256, 14.vii.1998, S.A. Grubbs, 10♂, 24♀ (WKU); Tonoloway Creek, Hancock, 39.6981, -78.1565, 3.viii.1996, S.A. Grubbs, 2♀ (WKU). **Massachusetts:** Hampshire Co., Ware River, Rte. 32, 3 km S Gilbertsville, 42.2847, -72.2161, 28.vi.2006, S.A. Grubbs, ♂ (WKU). **Michigan:** Allegan Co., Rabbit River, 10 km W Wayland, 42.6779, -85.7612, 19.vi.2006, S.A. Grubbs, ♂, ♀ (WKU); Benzie Co., [Platte River], Benzie State Park, 44.7183, -86.1165, 23.viii.1960, R.A. Scheibner, ♀ (MSUC-8317); Berrien Co., Dowagiac Creek, U.S. 31, Niles, 41.8452, -86.2621, 30.vii.1994, S.A. Grubbs, 5♂, 4♀, 2 nymphs (WKU), same but 7.vi.2001, S.A. Grubbs, 4♂, 2♀ (WKU); Dowagiac Creek, M-51, 2 km N Niles, 41.8643, -86.2416, 19.vi.2006, S.A. Grubbs, 8♂, 9♀ (WKU); Galien River, New Buffalo, 41.8084, -86.7154, 22.vi.1957, J. Lowe, 2♂, ♀ (CNC-500); Clare Co., Middle Branch Tobacco River, 11 km NE Clare, 43.9092, -84.7006, 24.vii.2006, S.A. Grubbs, ♂, 2 nymphs, exuviae (WKU); Clinton Co., Vermillion Creek, Bath, 42.8345, -84.3692, 15.vii.1955, R.L. Fischer, ♂ (MSUC-7832); Gladwin Co., Cedar River, 5 km N Beaverton, 43.9308, -84.4760, 24.vii.2006, S.A. Grubbs, ♀ (WKU); Hillsdale Co., East Branch St. Joseph River, 14 km W Hudson, Lost Nation State Game Area, 41.8635, -84.5145, 23.vi.2006, S.A. Grubbs, ♂, ♀ (WKU); East Fork West Branch St Joseph River, 14 km S Hillsdale at Dimmers Road, 41.7960, -84.6380, 27.vii.2000, J. Mandrelle, 5♂, 11♀ (OBS-6338); West Branch St. Joseph River, Boy Scout Camp Property, 5 km E State Rte. 49 at Territorial Road, 41.7071, -84.6903, 27.vi.2000, J. Mandrelle, ♂, 7♀ (OBS-6324); Ingham Co., [Grand River], Lansing, 42.7325, -84.5497, 23.vi.1981, ♂ (MSUC-2368); Isabella Co., Pine River, 8 km N Cedar Lake, Edmond State Game Area, 43.4733, -84.9701, 21.vi.2006, S.A. Grubbs, 3♀ (WKU); Kalamazoo Co., Kalamazoo River, 1.5 km SW Galesburg at 35th St., 42.2803, -85.4285, 19.vi.2010, R.E. DeWalt, M. Pessino and E.W. Hernandez, 3♂, ♀ (INHS-550553); Lake Co., Pere Marquette River, 7 km E Baldwin, 43.8869, -85.9370, 23.vii.2006, S.A. Grubbs, 2♂ (WKU); Lenawee Co., River Raisin, 10 km NE Adrian, 41.9492, -83.9415, 23.vi.2006, S.A. Grubbs, ♂ (WKU); [River Raisin], Clinton, 42.0718, -83.9796, 28.vi.1941, T.H. Frison and H.H. Ross, ♂ (INHSPle-3828); Livingston Co., Portage River, 3 km SE Hell at Dexter-Town Hall Rd, 42.4242, -83.9497, 9.vi.2010, R.E. DeWalt, M. Pessino and E.W. Hernandez, 12♂, 12♀ (INHS-514942); Menominee Co., Little Cedar River, 6 km W Carney at G18, 45.5799, -87.6332, 9.vii.2011, R.E. DeWalt, 2♀ (INHS-552590); Montcalm Co., Fish Creek, Carson City, 43.1773, -84.8566, 21.vi.2006, S.A. Grubbs, 3♂ (WKU); Flat River, Greenville, 43.1771, -85.2478, 21.vi.2006, S.A. Grubbs, ♂ (WKU); Flat River, at Hunter Lake, T11N R8W Sec. 33, 43.2921, -85.2592, 8.vii.1966, J. P. Donahue, 3♀ (MSUC-7480); Presque Isle Co., 45.3077, -83.8724, 5.viii.1948, ♂ (MSUC-7839); Saginaw Co., Bad River, 10 km W St. Charles, 43.2987, -84.2689, 21.vi.2006, S.A. Grubbs, ♂ (WKU); St. Clair Co., Bad River, 15 km SE Yale, 43.0926, -82.6181, 22.vi.2006, S.A. Grubbs, 3♂, 2♀ (WKU); St. Joseph Co., Pigeon River, 7 km WSW Pigeon River, 41.7764, -85.7213, 19.vi.2006, S.A. Grubbs, 3♂, ♀ (WKU), same but 21.vii.2006, S.A. Grubbs, ♂, ♀ (WKU); Portage River, Parkville, 42.0146, -85.5480, 19.vi.2006, S.A. Grubbs, ♂, 2♀ (WKU); Prairie River, Centreville, 41.9291, -85.5284, 21.vii.2006, S.A. Grubbs, ♀ (WKU); Washtenaw Co., Huron River 3 km SE Dexter, 42.3283, -83.8531, 22.vi.2006, S.A. Grubbs, 2♂, ♀ (WKU); Huron River, Scio township, 42.3235, -83.8404, 20.vi.1988, T.E. Moore, ♂, ♀ (UMMZ-2807). **Missouri:** St. Francois Co., Big River, Adjacent to picnic grounds, 37.9669, -90.5346, 19.vi.2008, R.E. DeWalt, ♂ (INHS-515558). **Ohio:** Ashland Co., Clear Fork [Mohican] River, SE Pleasant Hill Reservoir, 40.6082, -82.2540, ♀, T. Yamamoto, L. Kohalmi, 1.viii.1968, ROME-9773); Ashtabula Co., Ashtabula River, 1.9 km WNW Gageville, 41.8486, -80.6895, 15.vii.1998, M. Silvaggio, 5♀ (OBS-3047); Columbiana Co., Little Beaver Creek, Wayne Twp. [at Steubenville Rd], 40.6947, -80.7658, 14.vii.1997, E.G. Chapman, 2♀ (OBS-6653); Coshocton Co., Bucklew Run, 5 km E Warsaw at Twp. 28 Rd., 40.3323, -81.9378, 26.vi.1999, S.W. Chordas III and J. Thompson, 5♂, 2♀ (OBS-3212); Defiance Co., Mud Creek, 13 km WNW Defiance, 41.3339, -84.4968, 17.vii.2000, S.W. Chordas III and E.G. Chapman, ♀ (OBS-6372); Lake Co., Paine Creek, SE Lane, 41.7208, -81.1761, 22.vi.1999, ♀ (INHS-163045); Licking Co., North Fork Licking River, 3 km S Utica, 40.2095, -82.4428, 13.vii.1999, S. O’Dee, ♀ (OBS-2904); Miami Co., Rush Creek, 5 km NE Piqua, 40.1821, -84.2151, 17.vii.2000, S.W. Chordas III and E.G. Chapman, ♀ (OBS-3064); Paulding Co., Maumee River, 16 km NW Paulding, 41.1983, -84.7467, 17.vii.2000, S.W. Chordas III and E.G. Chapman, ♂, ♀ (OBS-6330); Pike Co., Pike State Forest, 39.1031, -83.2682, 1.vi.1938, C.R. Neiswander, 2♂, ♀ (INHSPle-3907); Richland Co., Rocky Fork Mohican River , SE Mansfield, 40.7025, -82.3666, 27.vii.1999, E.G. Chapman, 2♀ (OBS-6313); Scioto Co., 38.8371, -83.0445, 1.vi.1925, C.H. Kennedy, ♂ (OSU-10765); Vinton Co., Pike Run, at SR 327 deadend, 39.3074, -82.7315, 7.vii.1996, J. McCreery, ♀ (OBS-7229); Wayne Co., Killbuck Creek, 9 km SW Wooster, 40.6945, -81.9798, 22.vi.1999, E.G. Chapman, 8♀ (OBS-6327); Williams Co., East Branch St. Joseph River, 4 km N Pioneer, 41.6950, -84.5107, 27.vii.2000, J. Mandrelle, 3♀ (OBS-6339). **West Virginia:** Morgan Co., Cacapon River, Rte. 9, 39.5286, -78.3478, 12.vi.1998, S.A. Grubbs, 3♂, 3♀ (WKU). **Wisconsin:** Burnett Co., St. Croix River, Riverside, 46.0772, -92.2461, 25.vi.2010, R.E. DeWalt, E. Hernandez and M.M. Brown, 2♂, 3♀ (INHS-516406); Dane Co., 43.0186, -89.5498, 30.vi.1949, W. McNeel, ♀ (WIRC-7152); Fond Du Lac Co., T13N, R19E, S23, 43.6687, -88.1938, 24.vi.1975, ♀ (WIRC-14414); Jackson Co., Halls Creek, , 44.3594, -90.7845, 18.vi.1968, ♀ (WIRC-14729); Lincoln Co., Prairie River, WI-17, 45.3361, -89.4650, 20.viii.1992, R.W. Baumann and C.R. Nelson, ♂, ♀ (BYU-1); Marinette Co., Menominee River, 18 km NE Amberg at CR-Z, 45.5814, -87.7866, 26.vi. 2010, R.E. DeWalt, E. Hernandez and M.M. Brown, 10♂ (INHS-550141); Price Co., [South] Fork Flambeau River, Fifield, 45.8798, -90.4156, 18.vi.1934, T.H. Frison and C.O. Mohr, ♂ (INHSPle-3908); Vilas Co., Trout River, Trout Lake , 46.0350, -89.7059, 22.vii.1937, T.H. Frison and H.H. Ross, ♂ (INHSPle-3811).

**Figure 1.**
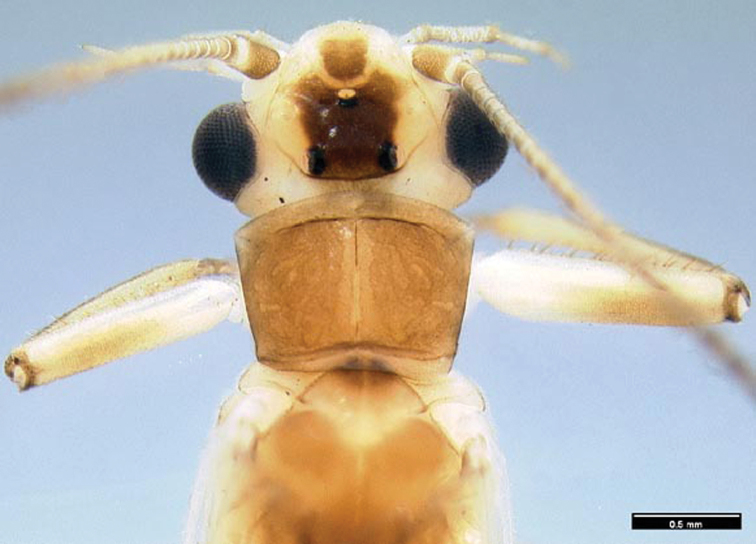
*Perlesta ephelida*. 1. Adult head and pronotum, dorsal.

**Figures 2–7.**
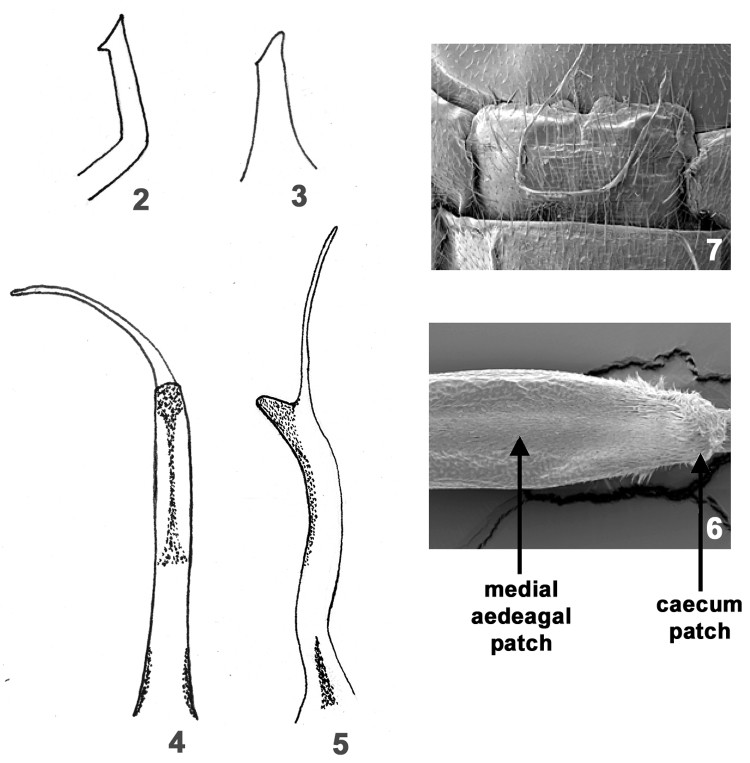
*Perlesta ephelida*. **2** Left paraproct, lateral **3** Left paraproct, caudal **4** Aedeagus, dorsal **5** Aedeagus, lateral **6** SEM micrograph, aedeagus, dorsal, 500X **7** SEM micrograph, female terminalia, ventral, 150X.

**Figures 8–13.**
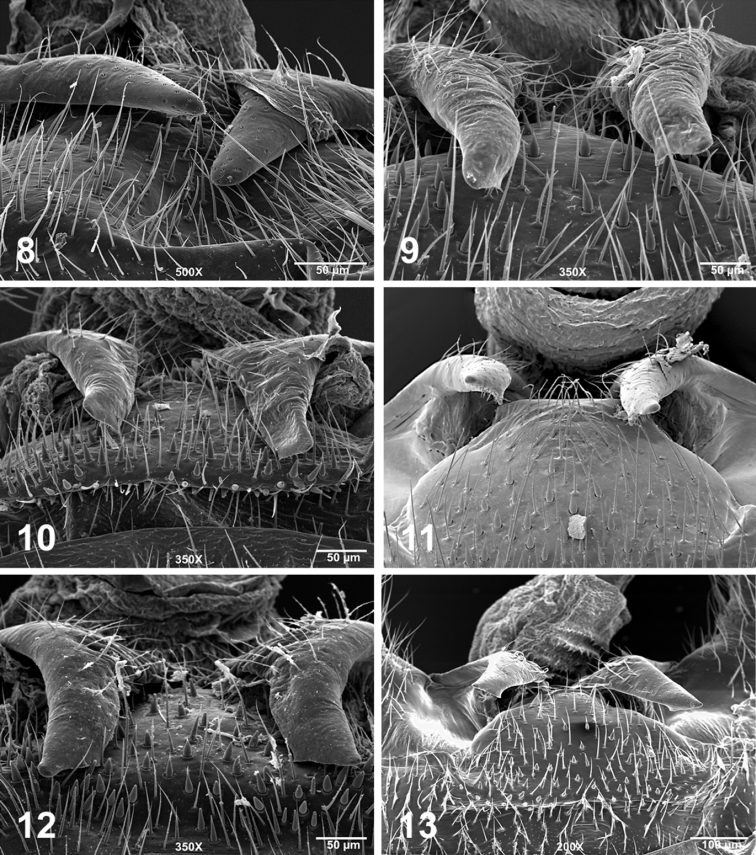
Male terminalia, posterodorsal view, SEM micrographs (**8** *Perlesta shubuta*, USA, Mississippi, Clarke Co., Rolling Creek, 16 May 2011, 500X **9–13** *Perlesta ephelida* **9** USA, Alabama, Clay Co., Enitachopco Creek, 18 May 2008, 350X **10** USA, Indiana, Bartholomew Co., East Fork White River, 11 June 2000, 350X **11** USA, Maryland, Washington Co., Licking Creek, 12 July 1997, 350X **12** USA, Michigan, Calhoun Co., South Branch Kalamazoo River, 25 July 2006, 350X **13** USA, Missouri, Oregon Co., Eleven Point River, 27 June 2011, 200X.

**Figures 14–21.**
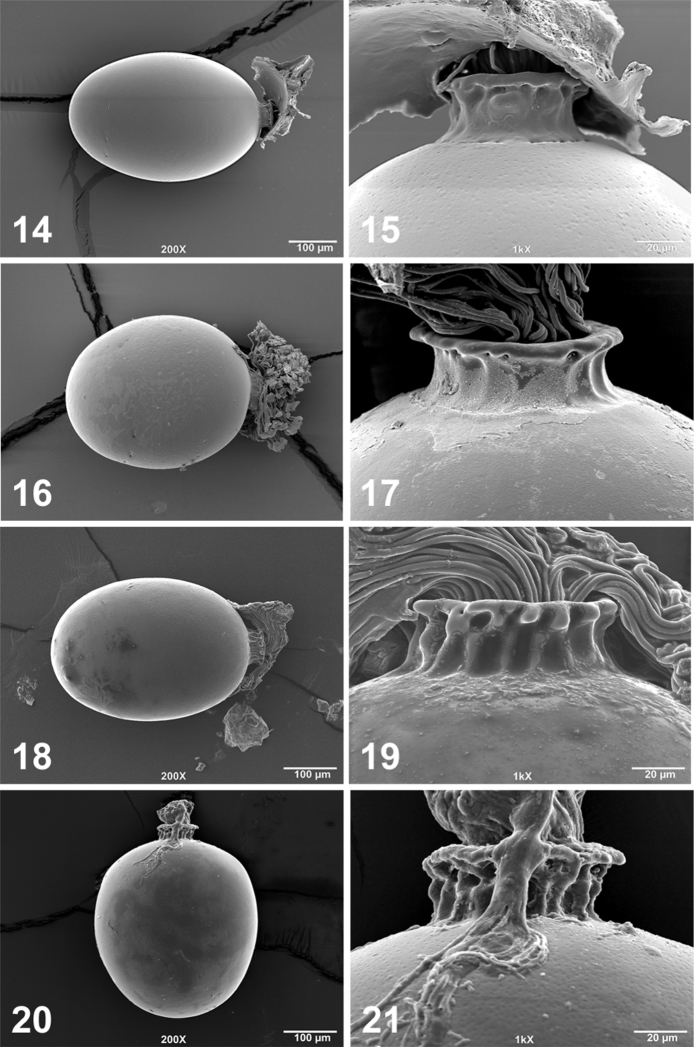
*Perlesta ephelida* egg SEM micrographs (**14–15** USA, Kentucky, Warren Co., Trammel Fork, Drakes Creek, 24 May 1999 **16–17** USA, Maryland, Washington Co., Licking Creek, 12 July 1997 **18–19** USA, Michigan, Calhoun Co., South Branch Kalamazoo River, 25 July 2006 **20–21** USA, Arkansas, Lawrence Co., Spring River, 27 June 2011) **14, 16, 18, 20** Entire egg, 200X; 15, 17, 19, 21. Anterior pole and collar, 1000X.

#### Adult habitus.

Head yellow with a dark brown subquadrate ocellar patch and a small, light brown subtriangular patch anterior to the anterior ocellus, pronotum golden-brown with a very faint lighter medial stripe in some specimens (Fig. 1). Wing membrane and veins amber except for pale costal region. Femora dorsally brown, laterally and ventrally yellow; tibia brown. Abdominal terga and sterna yellow. Cerci pale yellow proximally, brown distally.

#### Male.

Forewing length 7.5–8.5 mm. Tergum 10 mesal sclerites brown, sensilla basiconica patch distinct with interspersed long hairs (Figs 9–13). Paraproct broadest basally, apically acute, with distinct subapical tooth directed anteromedially (Fig. 2) and typically seen dorsally (Figs 9–13), in caudal view resembling a hypotenuse triangle and tooth visible (Fig. 3). Penis tube + sac long, caecum moderately-sized and thumb-shaped (Fig. 4), lateral sclerite distinct, dorsal patch broad basally, narrowing to narrow strip before expanding apically over caecum and tube (Fig. 5). At higher magnification the dorsal aedeagal patch hairs are noticeably shorter than the expanded portion over the caecum (Fig. 6).

#### Female.

Forewing length 9.0–11.0 mm. Subgenital plate lobes comprise medial half of 7^th^ sternite, extending scarcely past anterior margin of 8^th^ sternite, outer margins rounded, inner margins rounded and expanded slightly distally, separated by a shallow v-shaped notch (Fig. 7).

#### Egg.

Oval. Collar wide and distinctly stalked, highly-infolded, and flanged distally. Chorion smooth or finely pitted (Figs 14–21). Micropyles located ca. ¼ from posterior pole.

#### Nymph.

Undescribed.

#### Etymology.

The species name is a derivation of the Greek word (“ephelis”) for freckle (Brown 1956), in reference to the heavily-freckled head mask of the nymphs and associated exuviae.

#### Diagnosis.

The males of *Perlesta ephelida* will key to couplets 11 and 12 in Stark (2004), with a paraproct spine visible in lateral view and a group that includes *Perlesta shubuta* Stark, *Perlesta puttmanni* Kondratieff & Kirchner, 2003, and *Perlesta decipiens* (Walsh, 1862). Although the paraproct spine of *Perlesta ephelida* is directly somewhat anteriorly, it is mainly mesally-directed and typically seen in dorsal aspect (Figs 9–13), and superficially similar to *Perlesta puttmanni* (see Kondratieff and Kirchner 2003, Fig. 10). The paraproct spine of *Perlesta shubuta* is not visible in dorsal aspect and the tips are rounded apically (Fig. 8); the epiproct tips are pointed apically in *Perlesta ephelida* (Figs 9–13). The paraproct spine in *Perlesta puttmanni* is visible caudally (see Kondratieff and Kirchner 2003, Fig. 12), which is also seen in this aspect in *Perlesta ephelida* (Fig. 3), but is not visible in *Perlesta shubuta* (see Stark 2004, Fig. 7.352). The narrow aedeagal dorsal patch of *Perlesta ephelida* (Fig. 4) is slightly broader than *Perlesta puttmanni* (see Stark 2004, Fig. 7.367), whereas both *Perlesta shubuta* (see Stark 2004, Fig. 7.369) and *Perlesta decipiens* (see Stark 2004, Fig. 7.306) bear conspicuously broader patches. The egg of *Perlesta ephelida* possesses a wide, well-developed, and distally-flanged collar (Figs 15, 17, 19, 21), resembling *Perlesta decipiens* (Stark 2004, Fig. 7.397) but easily contrasted from the short collars of *Perlesta shubuta* (Figs 23–24), and *Perlesta puttmanni* (see Kondratieff and Kirchner 2003, Fig. 15). The subgenital plate of *Perlesta decipiens* (see Stark 2004, Fig. 7.379) has prominent truncate lobes and a deep v-shaped notch while the medial notch of *Perlesta ephelida* is shallow and v-shaped (Fig. 7). The female of *Perlesta ephelida* can only be confidently identified if mature eggs are present and associated with a male with a fully-extruded aedeagus.

**Figures 22–24.**
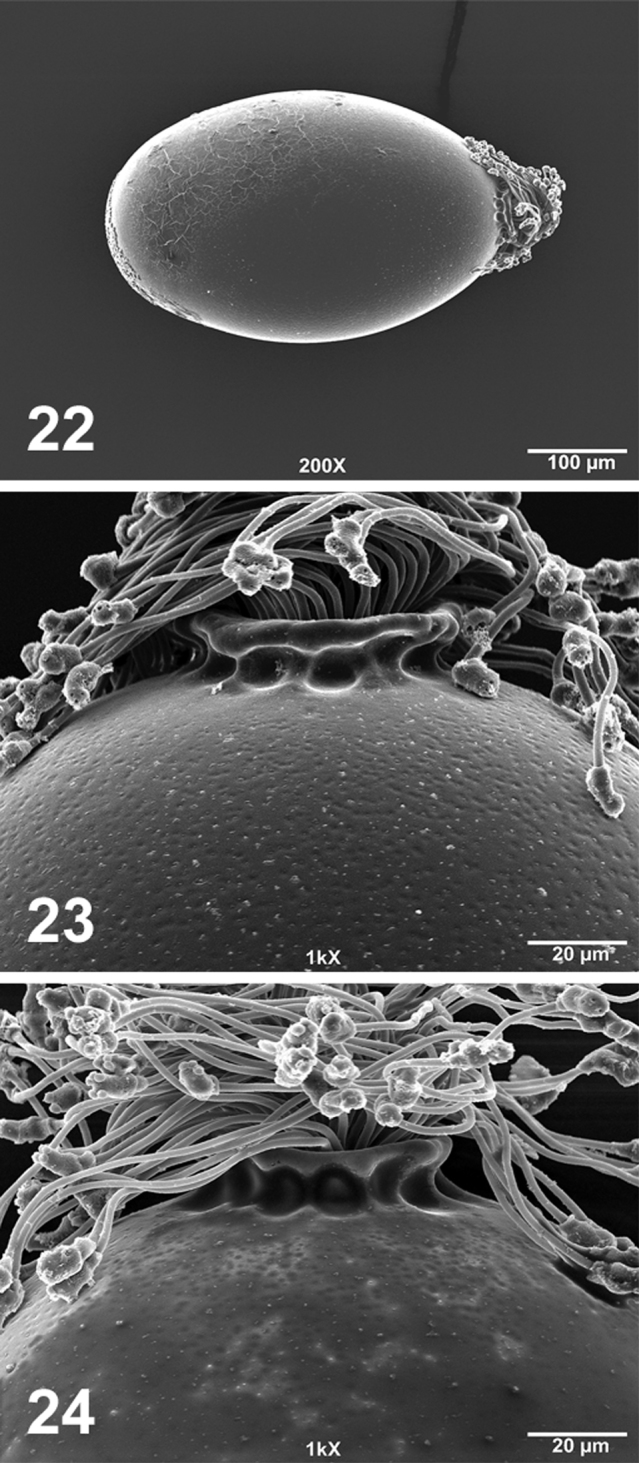
*Perlesta shubuta* egg SEM micrographs (**22–23** USA, Mississippi, Clarke Co., Rolling Creek, 16 May 2011 **23** USA, Alabama, Choctaw Co., Bogue Chitto Creek, 17 May 2011) **22** Entire egg, 200X **23–24** Anterior pole and collar, 1000X.

#### Remarks.

The type locality is a 5^th^-order gravel-bottom stream positioned in the Western Pennyroyal Karst Plain Level IV Ecoregion. Additional stonefly species obtained from the type locality were *Allocapnia granulata* (Claassen, 1924), *Allocapnia nivicola* (Fitch, 1847), *Allocapnia recta* (Claassen, 1924), *Allocapnia rickeri* Frison, 1929, *Amphinemura alabama* Baumann, 1996, *Allocapnia nigritta* (Provancher, 1876), *Strophopteryx fasciata* (Burmeister, 1839), *Taeniopteryx maura* (Pictet, 1841), *Acroneuria frisoni* Stark & Brown, 1991, *Perlinella drymo* (Newman, 1839), *Perlesta ephyre* (Newman, 1839), *Neoperla catharae* Stark & Baumann, 1978, *Neoperla stewarti* Stark & Baumann, 1978, *Clioperla clio* (Newman, 1839), *Isoperla decepta* Frison, 1935, and *Pteronarcys dorsata* (Say, 1823).

*Perlesta ephelida* is distributed broadly across the central and eastern U.S. from eastern Alabama northeast to Massachusetts, north to the Great Lakes region and Iowa, and northwest to the Interior Highlands region of Arkansas, Missouri, and Oklahoma. *Perlesta ephelida* is expected to be collected from intervening states (e.g. Pennsylvania, Tennessee) where this species has yet to be reported. In contrast, *Perlesta shubuta* appears to be restricted to a narrow latitudinal belt in the Gulf Coast region of the southeastern U.S., known currently from Louisiana east to northern Florida panhandle and likely to South Carolina.

The *Perlesta shubuta* records reported from Illinois (DeWalt et al. 2005, DeWalt and Grubbs 2011), Indiana (DeWalt and Grubbs 2011), Iowa (Heimdal et al. 2004), Michigan (Grubbs and Bright 2001), and Ohio (DeWalt et al. 2012) refer to *Perlesta ephelida*. Similarly, the *Perlesta decipiens* records from Calhoun (Terrapin Creek) and Clay (Enitachopco Creek) counties noted in Grubbs (2011) are now of *Perlesta ephelida*.

#### Modified key to couplets 11 and 12 in Stark (2004) to identify males of *Perlesta ephelida*:

**Table:** 

11	Dorsal aedeagal patch covers about 1/3 of tube surface length (Stark 2004, Fig. 7.369); forewing length 6–7m	*Perlesta shubuta* Stark
–	Dorsal aedeagal patch covers most of tube surface length (Stark 2004, Fig. 7.367); paraprocts forewing length 8–10 mm	12
12	Paraproct spine directed mesad, visible in caudal aspect (Stark 2004, Fig. 7.344)	12a
–	Paraproct spine directed forward, not visible in caudal aspect (Stark 2004, Fig. 7.310)	*Perlesta decipiens* (Walsh)
12a	Dorsal aedeagal patch narrower	*Perlesta puttmanni* Kondratieff & Kirchner
–	Dorsal aedeagal patch wider	*Perlesta ephelida* Grubbs & DeWalt, sp. n.

## Supplementary Material

XML Treatment for Perlesta shubuta

XML Treatment for Perlesta ephelida
